# Cognitive Training in Parkinson’s Disease Induces Local, Not Global, Changes in White Matter Microstructure

**DOI:** 10.1007/s13311-021-01103-9

**Published:** 2021-08-18

**Authors:** Chris Vriend, Tim D. van Balkom, Henk W. Berendse, Ysbrand D. van der Werf, Odile A. van den Heuvel

**Affiliations:** 1 Amsterdam UMC, Vrije Universiteit Amsterdam, Psychiatry, Amsterdam Neuroscience, De Boelelaan 1117, Amsterdam, Netherlands; 2 Amsterdam UMC, Vrije Universiteit Amsterdam, Anatomy and Neurosciences, Amsterdam Neuroscience, De Boelelaan 1117, Amsterdam, Netherlands; 3 Amsterdam UMC, Vrije Universiteit Amsterdam, Neurology, Amsterdam Neuroscience, De Boelelaan 1117, Amsterdam, Netherlands

**Keywords:** Structural connectivity, Diffusion, Parkinson’s disease, Neuroimaging, Cognitive training

## Abstract

Previous studies showed that cognitive training can improve cognitive performance in various neurodegenerative diseases but little is known about the effects of cognitive training on the brain. Here, we investigated the effects of our cognitive training paradigm, COGTIPS, on regional white matter microstructure and structural network topology. We previously showed that COGTIPS has small, positive effects on processing speed. A subsample of 79 PD patients (*N* = 40 cognitive training group, *N* = 39 active control group) underwent multi-shell diffusion-weighted imaging pre- and post-intervention. Our pre-registered analysis plan (osf.io/cht6g) entailed investigating white matter microstructural integrity (e.g., fractional anisotropy) in five tracts of interest, including the anterior thalamic radiation (ATR), whole-brain tract-based spatial statistics (TBSS), and the topology of the structural network. Relative to the active control condition, cognitive training had no effect on topology of the structural network or whole-brain TBSS. Cognitive training did lead to a reduction in fractional anisotropy in the ATR (*B* [*SE*]: − 0.32 [0.12], *P* = 0.01). This reduction was associated with faster responses on the Tower of London task (*r* = 0.42, *P* = 0.007), but this just fell short of our statistical threshold (*P* < 0.006). Post hoc “fixel-based” analyses showed that this was not due to changes in fiber density and cross section. This suggests that the observed effect in the ATR is due to training-induced alterations in neighboring fibers running through the same voxels, such as intra-striatal and thalamo-striatal fibers. These results indicate that 8 weeks of cognitive training does not alter network topology, but has subtle local effects on structural connectivity.

**Supplementary Information:**

The online version contains supplementary material available at 10.1007/s13311-021-01103-9.

## Introduction

Cognitive training has positive effects not only on cognitive performance but also on the brain, showing that cognitive training can increase neural efficiency and counteract aging- or disease-related neural dysfunction (van Balkom et al. 2020). Cognitive training may also change the microstructure of the white matter and structural connectivity. In healthy elderly, cognitive training increased the integrity (measured by fractional anisotropy (FA)) of the uncinate fascicle [1] and anterior thalamic radiation (ATR) [2]. The effects of cognitive training may even be evident after 12 months and associated with training-induced improvement in processing speed [3]. Others, however, found no effect of cognitive training on white matter integrity [4, 5], but studies have overall been small. So far, only one study has investigated the effects of cognitive training on white matter integrity in Parkinson’s disease (PD), showing no changes immediately after cognitive training [6], or at 1-year follow-up [7].

Compared with healthy controls, PD patients exhibit lower white matter integrity in the corpus callosum and cingulate and temporal regions [8]. The inferior longitudinal fascicle also seems to be particularly vulnerable to the PD pathology and is associated with cognitive deterioration [9]. Another study also showed that relative to healthy participants, white matter integrity progressively deteriorates from relatively intact in cognitive preserved PD patients, to widespread dysfunction in PD patients with mild cognitive impairment (MCI) and dementia, including the ATR, corpus callosum, and inferior longitudinal fascicles [10]. According to graph analyses, the topology of the structural connectome of PD patients is also less efficient and clustered compared with healthy controls [11–14], especially in PD patients with cognitive impairment [15]. No study has yet investigated the effect of cognitive training on the topology of the structural connectome in PD patients.

In the COGnitive Training In Parkinson Study (COGTIPS), we investigated the efficacy of a home-based online cognitive training [16]. We included 140 PD patients and acquired MRI scans in a subset of 85 PD patients to assess the effects of cognitive training on the function and structure of the brain. Here, we report on the effects of cognitive training on white matter microstructure and the structural connectome. Based on previous studies, we expected cognitive training to improve the white matter microstructure of the three different segments of the corpus callosum, the inferior longitudinal fascicle, and the ATR. We additionally hypothesized that cognitive training would improve the efficiency of the structural connectome to transfer information (measured as increased global efficiency) and improve the interconnectedness of neighboring brain regions (increased clustering coefficient).

## Methods

### Participants and Intervention

Participants (*N* = 140) were randomized (1:1) to an experimental cognitive training or an active control [16]. In both conditions, participants performed an 8-week online, home-based, computerized intervention (three 45-min sessions per week). The cognitive training condition consisted of 13 training games that focused on executive functions, processing speed, attention, and visuospatial functions and were adapted from the “Braingymmer” online platform (www.braingymmer.com, a product by Dezzel Media). The AC consisted of three low-threshold games primarily based on “crystallized intelligence” factors, i.e., solitaire, hangman, and trivia questions. A major difference between the conditions was the adaptive difficulty of the cognitive training games, while difficulty of the games in the active control condition stayed constant. See our methods paper for more information about the intervention [16].

We acquired an MRI scan from 85 participants. General trial inclusion criteria were (1) mild to moderately advanced idiopathic PD (Hoehn & Yahr stage < 4), (2) significant subjective cognitive complaints (PD Cognitive Functional Rating Scale score > 3), and (3) access to and proficiency in using a computer or tablet with internet. General exclusion criteria were (1) a Montreal Cognitive Assessment score < 22, (2) indications of current drug or alcohol abuse, (3) moderate to severe depressive symptoms, (4) an impulse control disorder, (5) psychotic symptoms except for benign hallucinations, or (6) a history of traumatic brain injury. Exclusion criteria for participation in the MRI study were (1) presence of metal in the body, (2) pregnancy, (3) difficulty lying still for 60 min, (4) a space-occupying lesion, or (5) significant vascular abnormalities (Fazekas > 1). This study was approved by the medical ethical committee of VU University medical center and performed in accordance with the Declaration of Helsinki. All participants provided written informed consent. The trial was registered at clinicaltrials.gov (NCT02920632).

### Image Acquisition

MRI scans were acquired on a GE 3.0 T Discovery MR750 (General Electric, Milwaukee, USA) with a 32-channel head coil at the Amsterdam UMC location VUmc. We acquired diffusion-weighted images with a multi-shell single-spin echo echo-planar imaging sequence (TR = 7350 ms, TE = 81 ms, 2.5 × 2.5 mm^2^ in-plane resolution with 56 slices of 2.5 mm; no gap) with 73 interleaved directions (25 *b* = 1000 s/mm^2^, 24 *b* = 2000s/mm^2^, and 24 *b* = 3000 s/mm^2^) and 7 non-diffusion-weighted volumes (*b* = 0 s/mm^2^). We additionally acquired a 3D T1-weighted structural magnetization-prepared rapid acquisition gradient-echo (MPRAGE) with scan parameters according to the ADNI-3 protocol [17]: TR = 6.9 ms, TI = 900 ms, TE = 3.0 ms, matrix size 256 × 256, 1 mm^3^ isotropic voxels. Patients followed the same protocol at both time points.

### Image Processing

A more detailed account of the (pre)processing pipeline is provided in the supplementary methods and the scripts are available from: github.com/chrisvriend/DWI_processing_COGTIPS. Diffusion images were denoised using the *dwidenoise* tool in MRtrix3 [18] and subsequently processed using EDDY [19] in FMRIB Software Library (FSL) version 6.0.1 [20]. We used EDDY QC for quality assessment [21] and additionally calculated the median sum of squared error of the b1000 tensor fit. These image quality measures (IQMs) were compared across time and groups using the nparLD package in R (version 4.0.2). DWI volumes were visually inspected for residual motion-related artifacts and deleted if necessary. Scans were excluded in case of > 3 volumes per shell with motion artifacts. We used FSL DTI-FIT to fit the tensor to the *b* = 1000 s/mm^2^ data to determine FA, mean diffusivity (MD), axial diffusivity (AD), and radial diffusivity (RD) [22]. We used DTI-TK to register the DWI scans to a common space [23] and subsequently performed tract-based spatial statistics (TBSS) [24] to investigate pre-to-post-intervention changes in the white matter microstructure of the genu of the corpus callosum, body of the corpus callosum, splenium of the corpus callosum, inferior longitudinal fascicle (ILF), and ATR. The corpus callosum ROIs were derived from JHU-ICBM labels, while the ILF and ATR were derived from the JHU-ICBM tracts (25%) atlas. We multiplied each tract with the skeletonized mask and extracted the median value of the FA, MD, AD, and RD in the tract. We performed multi-shell anatomically constrained (probabilistic) tractography with 100 million random white matter seeds to construct a tractogram for each participant in MRtrix3 [18]. SIFT2 was applied to improve the accuracy of the reconstructed fibers and reduce false positive connections [25, 26]. The resulting tractogram was converted to a 222 × 222 structural connectivity matrix with 208 cortical areas derived from the Brainnetome atlas and 14 individually segmented subcortical areas with FreeSurfer.

### Graph Measures

We calculated graph measures to determine the topology of the structural brain network. We calculated the global efficiency, modularity, and average clustering coefficient from individual connectivity matrices. Global efficiency is the inverse of the average path length and provides a measure for the ability of a network to integrate information [27]. The average clustering coefficient quantifies the segregation of nodes in a network, i.e., the tendency of the network to segregate into locally connected nodes to form a specialized subunit. Modularity measures how many modules a network can be divided into. Modules consist of nodes with stronger connections between them compared with nodes outside their module [27].

### Cognitive Measures

Participants performed—among other cognitive tests [16]—a self-paced version of the Tower of London (ToL) task on a laptop computer [28] and a paper-and-pencil version of the Stroop Color-Word Test (SCWT) [29]. These tests were performed on the same day as the MRI scans. The ToL covers various executive functions including planning, inhibition, and working memory [30] and consists of 100 pseudo-randomized trials with five difficulty levels (task loads S1 to S5) that are scored on accuracy and response time. The SCWT is an attention, processing speed, and executive function task [29] and requires the participants to read three cards with 100 items as fast as possible. Card I consists of columns with words (blue, red, yellow, green) while card II consists of squares with colors that participants need to name. Card III consists of the same words as card I (in another order) but the words are printed in congruent and incongruent colors, and participants need to name the color of the ink, while suppressing the tendency to read the word. The outcome measure of each card of the SCWT is the time to finish (in seconds). For the current analyses, we only considered card I (SCWT-I) where participants have to read words as a proxy for processing speed.

### Data Analysis

Multivariate mixed model analyses were performed with the four diffusivity measures in each ROI after training as dependent variables, defined condition (cognitive training or active control) as independent variable, and included pre-training diffusivity measures as covariates. Diffusivity measures were Z-transformed and MD and RD values were inverted to ensure that higher values on all four diffusivity measures signified better microstructural integrity. We added age, sex, and education level as nuisance covariates in separate adjusted models. We additionally performed exploratory whole-brain voxel-wise analyses on the diffusivity measures within a skeleton of the white matter using permutated (10,000) threshold-free cluster enhancement (TFCE) and family-wise error (FWE) correction (*P* < 0.05).

The network topological measures were analyzed with univariate linear mixed-models using network measures after training as outcome, the pre-training value as covariate, and condition as independent variable. Age, sex, and education level were added as nuisance covariates in separate models. The association between changes in DWI-derived measures (diffusivity or network topology) and training-induced changes in response time on the ToL task or SCWT card I were analyzed using repeated measures correlations (rmcorr package in R) [31]. Because of high interdependence between the diffusivity measures within each tract and topological measures, these correlations were corrected for multiple comparisons using a D/AP-Sidak adjustment to take into account the mutual correlation between outcome measures [32]. For the analyses on white matter microstructure, with an alpha of 0.05, 20 outcomes (four diffusivity values × five ROIs) and a mutual correlation coefficient of *r* = 0.31, the adjusted *P*-value was set at *P*_adj_ = 0.006 (determined using quantitativeskills.com/sisa/calculations/bonhlp.htm). For the topological analyses, the adjusted *P*-value was *P*_adj_ = 0.027 (alpha = 0.05, 3 outcomes, *r* = 0.44). We also explored the effect of the training on the connectivity strength between the default mode network (DMN), frontoparietal network (FPN), ventral attention network (VAN), and dorsal attention network (DAN) and their topology using the Yeo network parcellation [33]. Results on the subnetwork level were corrected for multiple comparisons using the false discovery rate (FDR; *q* = 0.05). The analysis plan was pre-registered at osf.io/cht6g and performed on the intention-to-treat sample only.

## Results

### Demographic and Clinical Characteristics

**Fig. 1:**
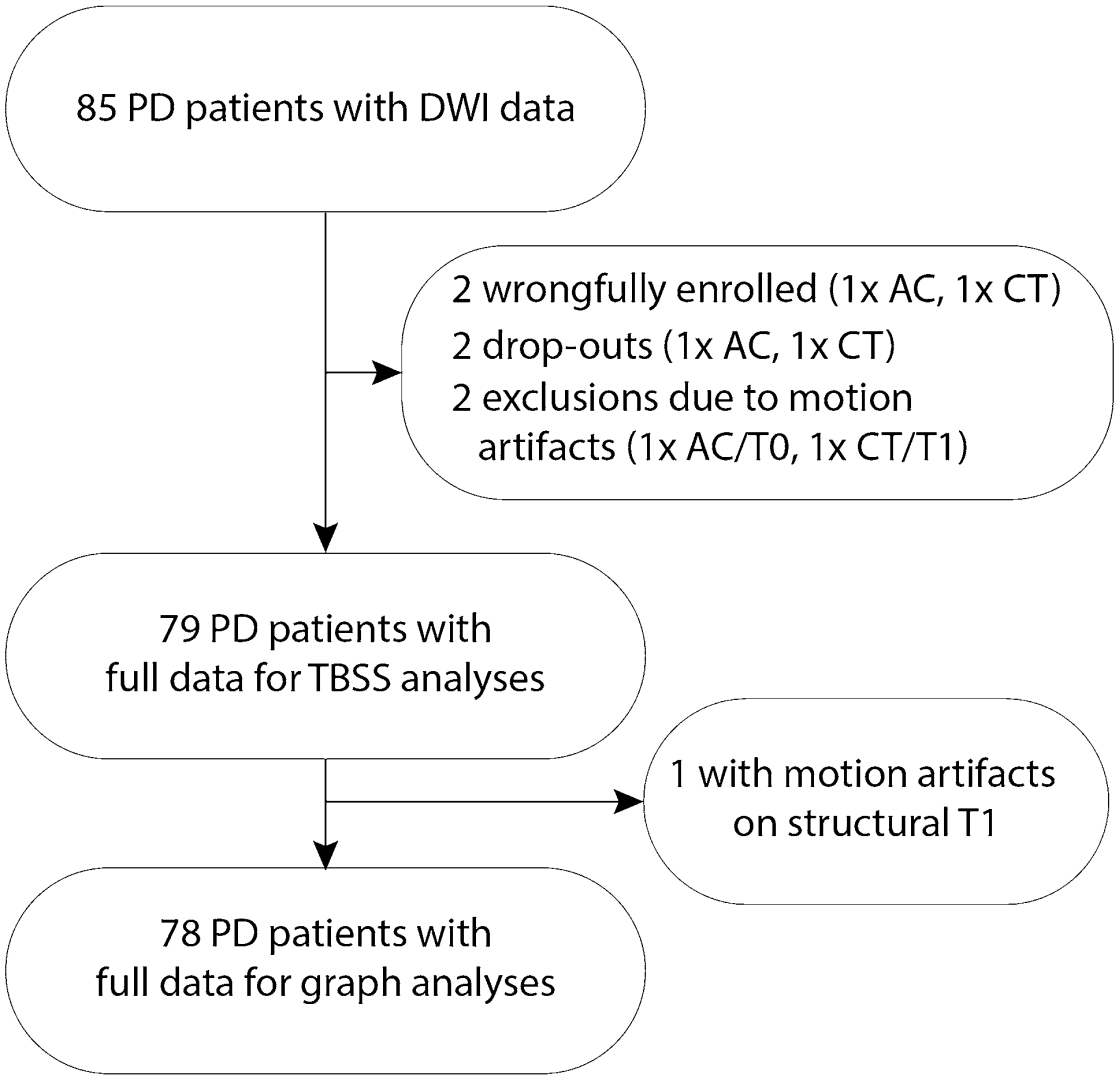
Flowchart. Abbreviations: AC, active control; EXP, experimental cognitive training group; TBSS, tract-based spatial statistics

From the original 85 PD patients with DWI data, six were excluded for the analyses of WM microstructure and one additional patient was excluded for the graph analysis (see flowchart in Fig. 1). Patients in both conditions were adequately matched on all demographic and clinical measures (see Table 1), except for PD-CFRS (*U* = 600.5, *P* = 0.02). Supplementary Table 1 shows the effects of the intervention on performance on the ToL and SCWT in this subsample of 79 PD patients. Compared with the full sample of PD patients (see [34]), we observed similar but not statistically significant effect sizes, likely due to the decrease in power.

**Table 1:** Demographic and clinical characteristics

	**Active control (*n* = 39)**	**Cognitive training (*n* = 40)**	**Statistics**
Sex (*N* (%))			
Male	26 (66.7%)	20 (50%)	χ^2^_(1) _= 2.3, *P* = 0.13
Female	13 (33.3%)	20 (50%)	
Age (years)	63.3 (6.4)	63.3 (8.1)	*t*_(77)_ = − 0.01, *P* = 0.99
Education (years)	17.0 (4.4)	15.6 (3.6)	*t*_(77)_ = 1.5, *P* = 0.14
Education classification (*N* (%))^†^			χ^2^_(4) _= 1.8, *P* = 0.78
3	-	1 (2.5%)	
4	3 (7.7%)	3 (7.5%)	
5	9 (23.1)	12 (30%)	
6	15 (38.5%)	12 (30%)	
7	12 (30.8%)	12 (30%)	
Disease duration (years, median [range])	4 [1–16]	4 [0–14]	*U* = 775, *P* = 0.96
UPDRS-III	20.3 (9.4)	20.6 (8.7)	*t*_(77)_ = 0.08, *P* = 0.94
Hoehn & Yahr stage (*N* (%))			χ^2^_(4)_ = 3.6, *P* = 0.47
1	2 (5.1%)	3 (7.5%)	
1.5	1 (2.6%)	5 (12.5%)	
2	19 (48.7%)	16 (40%)	
2.5	11 (28.2%)	12 (30%)	
3	6 (15.4%)	4 (10%)	
LEDD T0 (median [range])	700 [0–1790]	682 [80–1665]	*U* = 734.5, *P* = 0.66
Medication change (*N* (%))	8 (20.5%)	7 (17.5%)	χ^2^_(1)_ = 0.12, *P* = 0.73
LEDD T1 (median [range])	734 [0–1790]	749 [80–1530]	*U* = 736, *P* = 0.66
MoCA	26.1 (2.4)	26.5 (1.8)	*t*_(77)_ = − 0.68, *P* = 0.50
Global cognitive function classification (*N* (%))			χ^2^_(3)_ = 4.1, *P* = 0.25
Normal cognition	8 (20.5%)	12 (30%)	
Single-domain MCI	5 (12.8%)	7 (17.5%)	
Multi-domain MCI	17 (43.6%)	18 (45%)	
PD dementia	9 (23.1%)	3 (7.5%)	
BDI	8.6 (4.3)	7.9 (4.2)	*t*_(77)_ = 0.70, *P* = 0.49
QUIP-RS (median [range]; *N* = 75)	20.0 [0–44]	14.0 [0–60]	*U* = 550, *P* = 0.11
PAS	11.3 (7.3)	9.1 (6.2)	*t*_(77)_ = 1.4, *P* = 0.15
AS	14.0 (4.3)	13.0 (4.3)	*t*_(77)_ = 1.08, *P* = 0.28
Credibility-expectancy	31.4 (6.6)	33.1 (6.5)	*t*_(77)_ = − 1.1, *P* = 0.26
PD-CFRS (median [range])	10.0 [4–22]	7 [3.3–18.0]	*U* = 514.5, *P* = 0.009
Compliance (%, median [range])	100 [70.8–100]	99.8 [91.9–100]	*U* = 779.5, *P* = 0.99
T0-to-T1 interval (days)	64.2 (7.2)	63.8 (4.8)	*t*_(78)_ = − .28, *P* = .78

Data are presented as mean (SD) unless otherwise indicated

*AS* Apathy Scale, *BDI* Beck Depression Inventory, *PAS* Parkinson Anxiety Scale, *PD-CFRS*\ Parkinson’s Disease – Cognitive Functional Rating Scale, *LEDD* levodopa equivalent daily dosage, *MCI* mild cognitive impairment, *MoCA* Montreal Cognitive Assessment, *QUIP-RS* Questionnaire for Impulsive-Compulsive Disorders in Parkinson’s Disease – Rating Scale, *UPDRS* Unified Parkinson’s Disease Rating Scale. For pre-to-post intervention changes in the clinical and cognitive measures, see [34]

^†^According to Verhage education classification [51]

### Microstructure

**Fig. 2:**
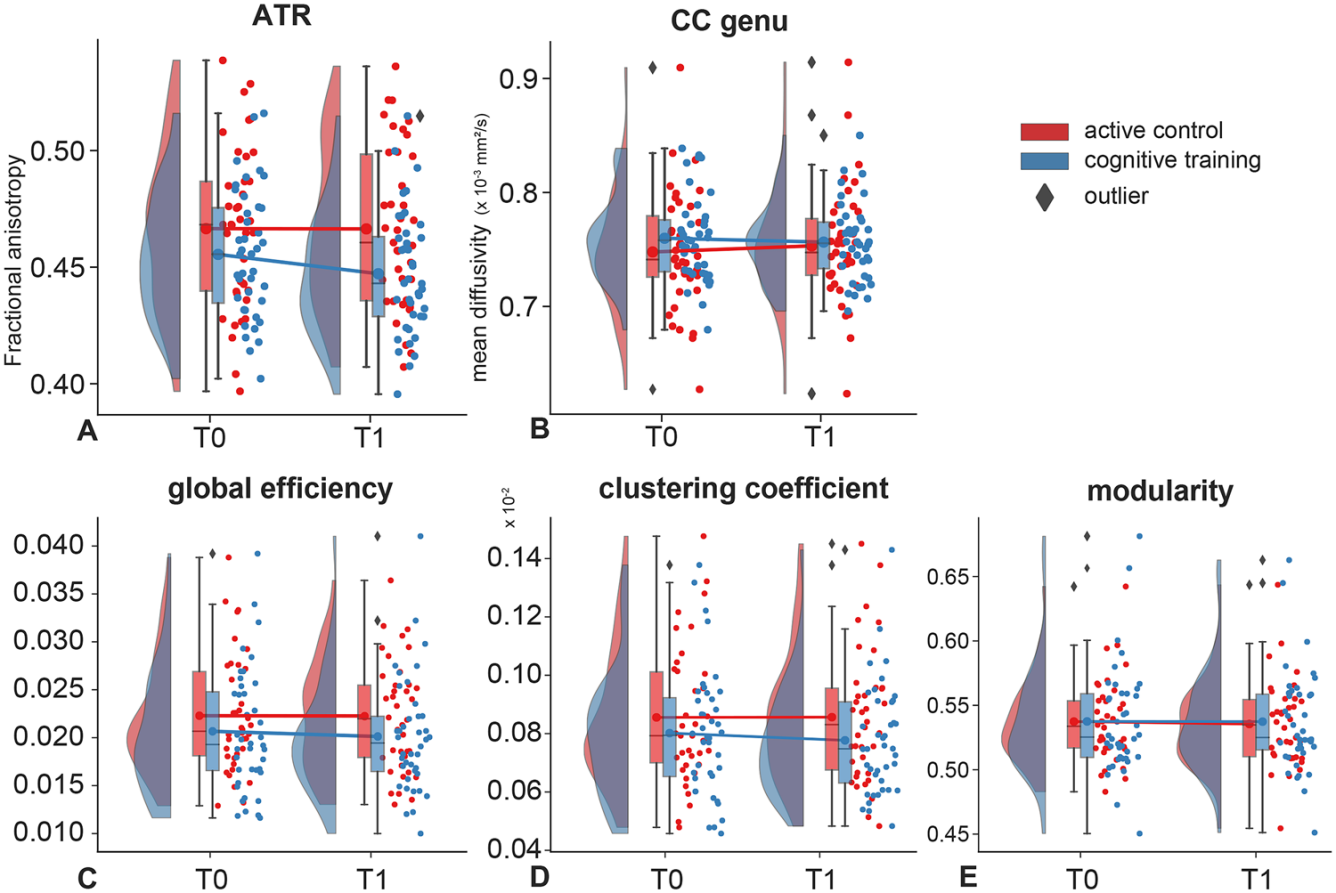
White matter microstructure and network topology. Raincloud plots of the effects of the intervention on diffusivity in the anterior thalamic radiation (**a**) and genu of the corpus callosum (**b**) and the network topological measures: global efficiency (**c**), clustering coefficient (**d**) and modularity (**e**). Abbreviations: ATR, anterior thalamic radiation; CC genu, genu of the corpus callosum

Results on the image quality measures are reported in the supplements. Overall diffusivity in the bilateral ATR was significantly lower in the cognitive training group compared with the active control group after training while adjusting for the microstructure at baseline (*B* [*SE*]: − 0.17 [0.08], 95% CI: − 0.32 to − 0.02, *P* = 0.03; see Table 2). This effect was driven by a reduction in FA in the cognitive training group after training (*B* [*SE*]: − 0.32 [0.12], 95% CI: − 0.45 to − 0.07, *P* = 0.01; see Fig. 2a). This effect remained significant after adjusting for age, sex, and years of education (*B* [*SE*]: − 0.29 [0.12], 95% CI: − 0.53 to − 0.05, *P* = 0.02). We also observed a significant difference in MD in the genu of the corpus callosum (*B* [*SE*]: 0.18 [0.09], 95% CI: 0.006 to 0.35, *P* = 0.04; Fig. 2b) but this effect was no longer significant after adjusting for covariates.

**Table 2:** Mixed model analyses of white matter microstructure

	**Baseline**		**T1**		**Group difference (crude model)**					Group difference (adjusted model)*				
	Active control *M* ± *SD*	CT *M* ± *SD*	Active control *M* ± *SD*	CT *M* ± *SD*	*B* [*SE*]	95% CI		*P*		*B* [*SE*]	95% CI		*P*	
**ATR**														
Overall diffusion					** − 0.17 [0.08]**	** − 0.32 to − 0.02**		**0.03**		− 0.15 [0.08]	− 0.30 to 0.006		0.06	
FA	0.467 ± 0.035	0.455 ± 0.028	0.466 ± 0.034	0.447 ± 0.027	** − 0.32 [0.12]**	** − 0.56 to − 0.07**		**0.01**		** − 0.29 [0.12]**	** − 0.53** to − **0.05**		**0.02**	
MD	0.685 ± 0.026	0.691 ± 0.027	0.682 ± 0.023	0.690 ± 0.020	− 0.20 [0.12]	− 0.44 to 0.04		0.10		− 0.18 [0.12]	− 0.42 to 0.06		0.15	
RD	0.488 ± 0.032	0.498 ± 0.032	0.486 ± 0.032	0.499 ± 0.027	− 0.19 [0.12]	− 0.43 to 0.05		0.12		− 0.17 [0.12]	− 0.41 to 0.07		0.20	
AD	1.086 ± 0.040	1.085 ± 0.045	1.082 ± 0.037	1.082 ± 0.040	0.02 [0.12]	− 0.22 to 0.25		0.90		0.04 [0.12]	− 0.20 to 0.28		0.75	
**ILF**														
Overall diffusion					0.008 [0.04]	− 0.08 to 0.10		0.85		− 0.003 [0.04]	− 0.09 to 0.08		0.93	
FA	0.472 ± 0.036	0.465 ± 0.028	0.473 ± 0.035	0.467 ± 0.031	0.02 [0.08]	− 0.13 to 0.18		0.77		0.01 [0.08]	− 0.14 to 0.17		0.89	
MD	0.760 ± 0.045	0.772 ± 0.033	0.759 ± 0.039	0.767 ± 0.031	0.06 [0.08]	− 0.10 to 0.22		0.46		0.05 [0.08]	− 0.11 to 0.20		0.56	
RD	0.542 ± 0.045	0.554 ± 0.034	0.541 ± 0.043	0.550 ± 0.035	0.06 [0.08]	− 0.10 to 0.21		0.47		0.05 [0.08]	− 0.11 to 0.20		0.56	
AD	1.185 ± 0.061	1.196 ± 0.051	1.184 ± 0.055	1.189 ± 0.048	− 0.11 [0.08]	− 0.26 to 0.06		0.19		− 0.12 [0.08]	− 0.27 to 0.04		0.14	
**CC genu**														
Overall diffusion					0.04 [0.06]	− 0.06 to 0.15		0.41		0.03 [0.06]	− 0.08 to 0.14		0.59	
FA	0.694 ± 0.078	0.697 ± 0.044	0.689 ± 0.072	0.693 ± 0.044	0.02 [0.09]	− 0.15 to 0.19		0.85		0.001 [0.09]	− 0.17 to 0.17		0.99	
MD	0.748 ± 0.052	0.761 ± 0.038	0.753 ± 0.051	0.757 ± 0.034	**0.18 [0.09]**	**0.006 to 0.35**		**0.04**		0.16 [0.09]	− 0.01 to 0.33		0.07	
RD	0.387 ± 0.076	0.395 ± 0.047	0.395 ± 0.074	0.396 ± 0.050	0.09 [0.09]	− 0.08 to 0.26		0.32		0.07 [0.09]	− 0.10 to 0.24		0.42	
AD	1.466 ± 0.116	1.491 ± 0.089	1.469 ± 0.101	1.479 ± 0.067	− 0.10 [0.09]	− 0.27 to 0.07		0.26		− 0.11 [0.09]	− 0.28 to 0.06		0.20	
**CC body**														
Overall diffusion					0.02 [0.06]	− 0.09 to 0.14		0.69		0.02 [0.06]	− 0.10 to 0.14		0.71	
FA	0.707 ± 0.056	0.710 ± 0.059	0.701 ± 0.060	0.706 ± 0.058	0.04 [0.09]	− 0.13 to 0.21		0.66		0.04 [0.09]	− 0.14 to 0.21		0.67	
MD	0.752 ± 0.058	0.756 ± 0.066	0.757 ± 0.062	0.755 ± 0.057	0.10 [0.09]	− 0.07 to 0.27		0.24		0.10 [0.09]	− 0.07 to 0.27		0.25	
RD	0.387 ± 0.069	0.389 ± 0.076	0.394 ± 0.071	0.392 ± 0.072	0.05 [0.09]	− 0.12 to 0.22		0.72		0.05 [0.09]	− 0.13 to 0.22		0.35	
AD	1.525 ± 0.118	1.535 ± 0.098	1.529 ± 0.120	1.528 ± 0.088	− 0.10 [0.09]	− 0.27 to 0.07		0.26		− 0.10 [0.09]	− 0.27 to 0.07		0.26	
**CC splenium**														
Overall diffusion					− 0.06 [0.09]		− 0.24 to 0.13		0.56	− 0.05 [0.10]		− 0.24 to 0.14		0.59
FA	0.832 ± 0.033	0.822 ± 0.037	0.828 ± 0.041	0.820 ± 0.037	− 0.02 [0.13]		− 0.29 to 0.24		0.85	− 0.02 [0.13]		− 0.28 to 0.24		0.88
MD	0.682 ± 0.026	0.687 ± 0.037	0.682 ± 0.026	0.689 ± 0.027	− 0.15 [0.13]		− 0.41 to 0.11		0.26	− 0.15 [0.13]		− 0.41 to 0.12		0.27
RD	0.236 ± 0.036	0.250 ± 0.047	0.239 ± 0.044	0.253 ± 0.043	− 0.08 [0.13]		− 0.34 to 0.18		0.55	− 0.08 [0.13]		− 0.34 to 0.19		0.57
AD	1.541 ± 0.080	1.534 ± 0.076	1.532 ± 0.083	1.529 ± 0.068	0.03 [0.13]		− 0.23 to 0.29		0.80	0.04 [0.13]		− 0.23 to 0.30		0.78

Significant differences are marked in bold

*CT* cognitive training, *FA* fractional anisotropy, *MD* mean diffusivity, *RD* radial diffusivity, *AD* axial diffusivity, *ATR* anterior thalamic radiation, *ILF* inferior longitudinal fascicle, *CC* genu genu of the corpus callosum, *CC* body body of the corpus callosum, *CC* splenium splenium of the corpus callosum

^*^Corrected for age, sex, and education in years

**Fig. 3:**
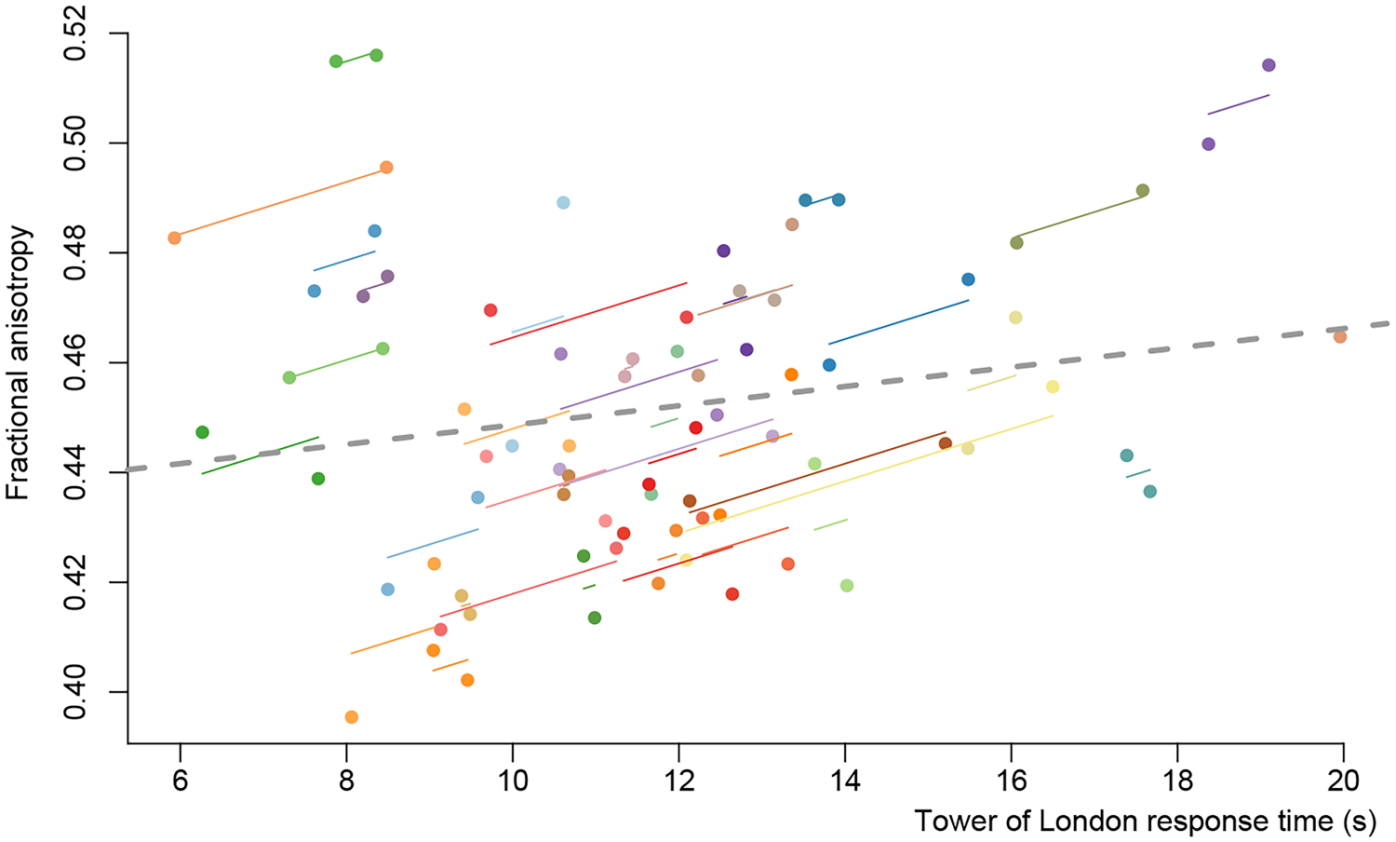
Repeated measure correlations. Correlation plot of the change in response time on the Tower of London from pre-training to post-training and change in median fractional anisotropy within the anterior thalamic radiation in the cognitive training group. Note that the FA in the ATR decreased in the CT group, alongside a decrease in (i.e., faster) response time

The other ROIs showed no significant effects (see also supplementary Fig. 2). When correlating the pre-to-post treatment changes in diffusivity with changes in cognitive performance, we observed a positive repeated measures correlation between changes in FA in the ATR in the cognitive training group and changes in ToL response time (*r* = 0.42, 95% CI: 0.12–0.66, *P* = 0.007; Fig. 3). This suggests that in the cognitive training group responses on the ToL are faster when FA decreases. Nevertheless, this correlation just fell short of our multiple comparisons correction (*P*_adj_ = 0.006).

### Network Topology

Cognitive training had no effect on whole-brain topology, relative to the active control condition (see Table 3 and Fig. 2c–e and supplementary results) and there were no correlations with pre-to-post intervention changes in cognitive performance.

**Table 3:** Mixed model analyses of global network topology

	**Baseline**		**T1**		**Group difference (crude model)**			Group difference (adjusted model)*		
	Active control *M* ± *SD*	Cognitive training *M* ± *SD*	Active control *M* ± *SD*	Cognitive training *M* ± *SD*	*B* [*SE*]	95% CI	*P*-value	*B* [*SE*]	95% CI	*P*-value
Global efficiency	0.022 ± 0.006	0.021 ± 0.006	0.022 ± 0.006	0.020 ± 0.006	− 0.0009 [< 0.001]	− 0.002 to 0.0007	0.26	− 0.001 [0.0007]	− 0.002 – 0.0004	0.16
Average clustering (× 10^−3^)	0.86 ± 0.24	0.80 ± 0.22	0.86 ± 0.22	0.78 ± 0.19	− 4.3 × 10^−5^ [3.2 × 10^−5^]	− 0.0001 to 2.1 × 10^−5^	0.18	− 4.8 × 10^−5^ [3.0 × 10^−5^]	− 0.0001 to 1.2 × 10^−5^	0.11
Modularity	0.54 ± 0.03	0.54 ± 0.04	0.54 ± 0.03	0.54 ± 0.04	0.002 [0.004]	− 0.005 to 0.009	0.62	0.003 [0.004]	− 0.004 to 0.010	0.42

*SE *standard error, *CI *confidence interval

^*^Corrected for age, sex, and education in years

### Explorative Analyses

Whole-brain TBSS analyses showed no differences in the white matter microstructure between the two conditions. Explorative analysis of the topology of neurocognitive subnetworks (i.e., efficiency and clustering) and the connections between them also did not reveal any significant effects of training (see supplementary Table 2).

### Post Hoc Analyses

We performed post hoc analyses to follow up on the observed effects of cognitive training on overall diffusivity—and particularly FA—in the ATR. The effect of training on overall diffusivity was similar across the left and right ATR, although only the left ATR showed a reduction in FA in the cognitive training group (*B* [*SE*]: − 0.33 [0.12], 95% CI: − 0.56 to − 0.09, *P* = 0.008) that remained after adjusting for covariates (*B* [*SE*]: − 0.30 [0.12], 95% CI: − 0.54 to − 0.06, *P* = 0.01). We additionally performed a post hoc “fixel” analysis on the ATR to better understand the unexpected decrease in FA in the cognitive training group. Fixels are *specific* fiber populations within a voxel [35, 36]. See supplementary methods for more details. These analyses showed that there were no effects of training on the fiber density or cross section of the ATR (supplementary Fig. 3 and supplementary Table 3). This suggests that the observed reduction in FA in the cognitive training group is not due to changes in density of cross section of fibers of the ATR.

## Discussion

This study investigated the effects of our online cognitive training program, COGTIPS [16], on WM microstructure and topology of the structural connectome. We showed that in the cognitive training group, relative to an active control condition, overall diffusivity within the ATR decreased, which was driven by a reduction in FA, while MD in the genu of the corpus callosum increased. Only the FA reduction in the (left) ATR in the cognitive training group remained significant after correcting for covariates. Interestingly, the decrease in FA in the ATR was associated with faster responses on ToL task (although this fell just short of our multiple comparison threshold). Training-induced faster responses on the ToL task were the main finding on the behavioral level of our randomized controlled trial [34]. Cognitive training had no effect on network topology on neither the global or subnetwork level.

The ATR is a major fiber bundle connecting the anterior and midline nuclei of the thalamus with the prefrontal cortex [37] and is therefore critically involved in the associative cortico-striatal-thalamo-cortical (CSTC) circuit and its associated functions [38, 39]. Multiple studies have shown dysfunction of the associative CSTC circuit in PD, particularly in relation to cognition [40–42]. Based on this, we hypothesized that cognitive training would increase FA relative to the active control group. Surprisingly, our results show an opposite pattern. FA measures the degree of diffusion within a single direction and a decrease in FA may therefore signify either lower axonal density (e.g., due to demyelination and increased free-water diffusion) or a higher proportion of crossing fibers (i.e., diffusion along multiple fibers). Standard tensor models cannot disentangle these two possibilities [43] due to averaging the diffusivity of multiple (crossing) fiber populations inside a voxel. We therefore performed a post hoc analysis of “fixels”, i.e., specific fiber populations *within* a voxel [35]. Fixel-based analysis is a relatively new method that allows the quantification of the density and cross section of specific tracts, even in voxels that contain crossing fibers [36]. This analysis showed that cognitive training did not alter the density or cross section of the ATR. The ATR runs through the anterior limb of the internal capsule that also contains other fibers. Most of these fibers run in parallel to the ATR (e.g., the superolateral medial forebrain bundle and frontostriatal fibers) [44], but others run perpendicular (i.e., fibers between the caudate nucleus, putamen, pallidum, and thalamus; see also supplementary Fig. 4). We therefore speculate that the decrease in FA in the cognitive training group is not due to a higher demyelination of the ATR, but due to a higher incidence of crossing fibers within the same voxels that connect these subcortical structures. Unfortunately the resolution of our scans limits our ability to confirm this and necessitates studies at ultra-high field strength [44]. Other analytical methods that rely on multi-shell DWI data, such as neurite orientation dispersion and density imaging (NODDI) [45], can also provide additional information on the effects of cognitive training on white matter microstructure.

Our results also showed that the reduction in FA in the ATR in the cognitive training group was associated with faster responses on the ToL task. Although this subsample of PD patients with a DWI scan did not show significant training-related increases in cognitive performance on the group level, our analyses in the entire sample of 140 PD patients showed positive effects of cognitive training on ToL response time, especially for the more cognitively demanding task load 4 [34]. Combining these results they suggest training-induced improvement in processing speed during executive functioning that is accompanied with an increase in intra-striatal or thalamo-striatal fibers on the individual level. Although there is some evidence for a role for thalamo-striatal connections in attention [46], and thalamo-striatal connections originating from the centromedian and parafascicular nucleus are particularly prone to PD-related neurodegeneration [47], the (plastic) effects of cognitive training on these connections are currently unknown.

The only other but smaller (*N* = 30) study that has investigated the effects of a 3-month cognitive training on white matter microstructure in PD patients showed no changes using a whole-brain TBSS approach [6]. Other studies on the effects of cognitive training on white matter microstructure seem to have exclusively been performed in healthy elderly populations and have produced mixed results [1–5]. It must be noted that the sample sizes of these studies have overall been small, the studies used different training paradigms, and the findings in healthy elderly may not readily be extrapolated to PD or other brain disorders. Interestingly, a recent study in 60 healthy elderly on the effects of a 2-year multi-domain training involving cognitive training, diet, exercise, and vascular risk management also observed widespread *decreases* in FA in the intervention group relative to the control group [48]. This unexpected finding was mainly seen in left-sided parietal, callosal, and subcortical fibers, including a segment of the ATR, and partly mirrors our own findings. They interpreted the decrease in FA, however, as an intervention-induced reversal of astrocytic hypertrophy and axonal swelling. Because our fixel-based analysis showed no training-induced changes in the fiber density or cross section of the ATR, this interpretation is, however, less likely for our findings.

Cognitive training had no effect on the structural connectome, either at the global or subnetwork level. In fact, network topology remained remarkably stable over time, with test–retest intra-class correlations (*ICC*) > 0.85 for the three global measures. To the best of our knowledge, only one previous study has investigated the effects of a cognitive training on the structural connectome [49] and none has been performed in PD patients. It is possible that the effects of cognitive training are limited to the regional level and do not generalize to global topological changes, at least not within a timeframe of 8 weeks. Indeed, Roman and colleagues showed that cognitive training had no effect on global network topology. Conversely, using network-based statistics (NBS) [50], they observed a significant group × time interaction effect on efficiency and strength of a subnetwork that involved connections between several brain areas in the temporal, frontal, parietal, and insular cortices as well as subcortical areas. NBS is a statistical approach that identifies a subnetwork based on between-group differences in edge strengths. It is therefore specific to a particular dataset and the identified subnetwork may not necessarily obey the normal hierarchical structure of the brain’s functional systems, such as the ones we investigated here using the Yeo parcellation [33].

A limitation of our study is the lack of a healthy control group that impeded us from assessing the severity of deviating white matter microstructure or topology in our PD patients before training or to assess the potential normalizing effects of cognitive training. Second, although there were no interaction effects for any of the image quality measures, the higher sum of squared error in the active control group may have affected some of our results as the sum of squared error represents the accuracy of the tensor fit (see supplementary results). The lack of an MRI session at a follow-up assessment also impeded us from investigating the longevity of the training-induced changes. Last, this subsample of PD patients with imaging data was insufficiently powered to detect differences in cognitive performance (see [16]). We did, however, observe similar effects sizes as we did for the full sample. The direction of the repeated measures correlation between ToL response time and FA in the ATR is also consistent with the effects observed at the group level, bolstering our findings. Strengths of this study are the large sample size and low attrition, rigorous quality control, and a description of the IQMs as well as the use of state-of-the-art registration (DTI-TK) and tractography (MRtrix3) algorithms and the use of a multi-shell DWI sequence to better deal with crossing fibers.

In conclusion, in this largest study on cognitive training in PD patients to date, we showed that our 8-week cognitive training program, COGTIPS, induces changes in local white matter microstructure that also correlate with cognitive improvement, but has no effect on the topology of the structural connectome at higher levels of organization. Because our post hoc “fixel” analyses showed no effect on fiber density or cross section, we speculate that the observed changes are due to changes in neighboring (crossing) fibers. These results suggest that cognitive training has subtle and only local effects on structural plasticity.

## Supplementary Information

Below is the link to the electronic supplementary material.

Supplementary file1 (PDF 1534 KB)

Supplementary file2 (DOCX 1392 KB)
